# Changes in inoculated *Salmonella enterica* subsp. *enterica* Serovar Enteritidis and other microbiological qualities of vacuum-packed carrot slices after treatment with aqueous extract of *Lobularia maritima*

**DOI:** 10.1016/j.heliyon.2024.e29065

**Published:** 2024-03-30

**Authors:** Anis Ben Hsouna, Natália Čmiková, Boutheina Ben Akacha, Rania Ben Saad, Wissem Mnif, Stefania Garzoli, Miroslava Kačániová

**Affiliations:** a Laboratory of Biotechnology and Plant Improvement, Centre of Biotechnology of Sfax, P.O. Box 1177, Sfax, 3018, Tunisia; b Department of Environmental Sciences and Nutrition, Higher Institute of Applied Sciences and Technology of Mahdia, University of Monastir, Monastir, 5000, Tunisia; c Institute of Horticulture, Faculty of Horticulture and Landscape Engineering, Slovak University of Agriculture in Nitra, Tr. A. Hlinku 2, 949 76, Nitra, Slovak Republic; d Department of Chemistry, College of Sciences at Bisha, University of Bisha, P.O. Box 199, Bisha, 61922, Saudi Arabia; e Department of Chemistry and Technologies of Drug, Sapienza University, P. le Aldo Moro, 5, 00185, Rome, Italy; f School of Medical & Health Sciences, University of Economics and Human Sciences in Warsaw, Okopowa 59, Warszawa, 01 043, Poland

**Keywords:** *Lobularia maritima*, Antimicrobial activity, *Salmonella enterica*, Carrot, Aqueous extract

## Abstract

After harvesting, pathogens can infect fresh vegetables in different ways. Pathogenic bacteria associated with fresh vegetables can cause widespread epidemics associated with foodborne illness. The aim of this study was to assess the microbiological quality of carrot slices after treatment with aqueous extracts of *Lobularia maritima* (AE*Lm*) at different concentrations AE*Lm*1 (10 mg/mL), AE*Lm*2 (5 mg/mL), AE*Lm*3 (2.5 mg/mL) and AE*Lm*4 (1.25 mg/mL), and *Salmonella enterica* subsp. *enterica* serovar Enteritidis, along with vacuum packaging and storage of carrots for 7 days at 4 °C. On days 1. and 7., total viable counts (TVC), and coliforms bacteria (CB), and *Salmonella* count were all analysed. Microorganisms that were obtained from carrots were identified using MALDI-TOF MS Biotyper Mass Spectrometry. The total viable, coliform bacteria and *Salmonella* counts were varied by the group of treatment. Higher counts were found in the control group on both days. The most isolated species of bacteria were *Salmonella enterica* and *Pantoea agglomerans* on the 1. day and *Klebsiella oxytoca* on the 7. day. The current study adds useful information for a better understanding of how *Salmonella enterica* reacts to the effect of AE*Lm* and its potential use as a sustainable washing method to eliminate bacteria from freshly cut carrots.

## 1 Introduction

Carrots can be contaminated with microbes from soil, water, transport and handling during processing. According to reports, microbiological studies focus on total mesophilic bacteria, lactic acid bacteria, enterobacteria or coliforms and psychrotrophic bacteria [[1], [2], [3], [4]]. In particular, on the day of processing, total aerobic mesophilic bacteria generally range between 4.4 and 5.9 log (Colony Forming Units) CFU/mL, whereas microorganisms range from 2.8 to 4.8 log CFU/mL and 1.9 to 4.6 log CFU/mL [[1], [2], [3]].

Studies on methods to extend food shelf life can be divided into two categories: either a comprehensive evaluation of quality is carried out, taking into account a variety of microbial groups, physicochemical variables and sensory quality, or the study is focuses on foodborne pathogens, which can grow in food, and which consequently can lead to safety problems. Results of interventions aimed at controlling reference strains of *Escherichia coli*, *Salmonella Typhimurium* or *Listeria monocytogenes* are often reported for carrot-based foods [[5], [6], [7], [8]]. *Salmonella* can be a risk because carrots are grown in the soil and are in frequent direct contact with the soil. The likelihood of foodborne disease is higher for ready-to-eat carrot slices that are processed in unorganised industries [9].

The occurrence of *Salmonella* spp. in plants is associated with the greatest risk of contamination in the food industry. In addition, strains of this genus form biofilms on many surfaces of processing equipment that come into contact with food, leading to cross-contamination of vegetables [10,11]. Based on washing and disinfection processes using mainly chemical disinfectants, effective intervention procedures are available to minimize Salmonella infections [12,13]. However, there are a number of risks and sustainability issues associated with these chemicals. The food industry should reduce or even eliminate the use of these chemical disinfectants therefore it is crucial to develop washing procedures using efficient “green washing solutions” [14]. In each of these investigations, the carrot meal is first sterilized or decontaminated, then the microbial target is injected, and finally the desired treatment is applied. There are not many carrot food preservation studies that directly address bacteria associated with spoilage [15,16].

In Northern Africa (Tunisia) and Southern Europe, the halophyte *Lobularia maritima* (*Alyssum maritimum*, Brassicaceae) is considered a potential environmental weed. It can grow in agricultural areas but is most commonly found on sandy beaches and dunes [17,18]. This plant has antioxidant, antibacterial, anti-inflammatory, and anticancer properties and therefore may be useful in the food industry and in the prevention of various diseases [17,19]. In industrial cooking, the young leaves, stems and flowers of *L. maritima* are used to flavour salads and other foods where spiciness is required. Natural antibacterial agents such as *L. maritima* are becoming increasingly popular as a label-friendly replacement for artificial food preservatives [17]. Antibacterial properties of plant extracts *in vitro* have been studied in detail. There *in situ* efficacy is less studied, probably due to the reduced efficacy of plant extracts in food [20]. Due to the complex and heterogeneous nature of the food environment, it cannot be guaranteed that *in vitro* results can be extrapolated to food products at concentrations that preserve their antibacterial efficacy without compromising their sensory properties. It is therefore necessary to assess their antimicrobial profile *in situ* and to seek other approaches to maximise their efficacy, including mixing of different treatments, so that they can be systematically applied in food matrices [20].

Considering the above, the present study was mainly aimed at screening the *in situ* antimicrobial activity of aqueous extracts of *Lobularia maritima* plant to enhance the safety of vacuum packed carrot slices against *Salmonella enterica*. The second part focused on the identification of bacterial species naturally occurring on carrot preparations during storage using mass spectrometry. This is the first study to use an aqueous extract of *L. maritima* on the viability of *S. enterica* inoculated on vacuum-packed carrots.

## 2 Material and method

### 2.1 Plant material and preparation of extracts

Aboveground plant parts of *L. maritima* plants were collected in March 2020 from the Chebba region (Mahdia, Tunisia, latitude 35.23°, longitude 11.11°) and air-dried at 25 °C for 2 weeks, prior to maceration. The protocol for the preparation and extraction of aqueous extract (AE) from *L. maritima* is briefly described in our previously published work [17].

There are several commonly used methods in the extraction and separation of medicinally active portions of plants. These include classes of preparations known as (i) Maceration, (ii) Infusion, (iii) Digestion, (iv) Decoction, (v) Percolation, (vi) Hot Continuous Extraction (Soxhlet), (vii) Microwave-assisted extraction, (viii) Aqueous Alcoholic Extraction by Fermentation, (ix) Counter-current Extraction, (x) Ultrasound-assisted extraction (Sonication), (xi) Supercritical Fluid Extraction, (xii) Phytonics Process. In this work we used the first class as described in the study of Ben Hssouna et al. study [17]. Maceration is an old method used to prepare medicines. It is considered a suitable method for effective and inexpensive drugs. In addition, it is an ideal technique for certain substances that are very insoluble in solvent and require only prolonged contact with the solvent.

### 2.2 Samples

Carrot (*Daucus carota*) samples were used in this study. The carrots were purchased from a legitimate retailer. The carrot samples were sent to the microbiological laboratory in a sterile refrigerator under hygienic conditions. Samples were transported from the authorized store to the laboratory within 10 min. A total of 0.5 kg of carrots were collected. The carrot samples were then cut into 5 g pieces with a sterile knife and each piece was weighed. A total of 60 5-g samples were prepared. The 5 g carrot samples were then treated with 100 μL of aqueous extract of *Lobularia maritima* (AE*Lm*1- AE*Lm*4) and vacuum packed using a Concept, Choce, Czech Republic vacuum packer. Every carrot sample (5 g) was packaged separately. The control sample groups included both vacuum-packed and unpackaged samples. The AE*Lm*1 was obtained according to the protocol described by Hsouna et al. [17] with an initial concentration 10 mg/mL. The residue obtained was stored at −20 °C in the dark.

The total samples of carrot slices were examined using different concentrations of aqueous extract of *Lobularia maritima* obtained by dilution of the mother solution such as AE*Lm*1 (10 mg/mL), AE*Lm*2 (5 mg/mL), AE*Lm*3 (2.5 mg/mL) and AE*Lm*4 (1.25 mg/mL). The samples were made in the following manner:

1. control aerobically packaged group: carrot samples were packed in polyethylene bags under aerobic conditions and stored at 4 °C;
2. control group with vacuum packaging: samples of fresh carrots were packed in polyethylene bags and stored under anaerobic conditions at 4 °C;
3. vacuum-packaged carrots were treated with AE*Lm*1, packaged in polyethylene bags, and stored under anaerobic conditions at 4 °C;
4. vacuum-packaged carrots were treated with AE*Lm*1+*Salmonella enterica* subsp. *enterica* serovar Enteritidis, packaged in polyethylene bags, and stored under anaerobic conditions at 4 °C;
5. vacuum-packaged carrots were treated with AE*Lm*2, packaged in polyethylene bags, and stored under anaerobic conditions at 4 °C;
6. vacuum-packaged carrots were treated with AE*Lm*2+*Salmonella enterica* subsp. *enterica* serovar Enteritidis, packaged in polyethylene bags, and stored under anaerobic conditions at 4 °C;
7. vacuum-packaged carrots were treated with AE*Lm*3, packaged in polyethylene bags, and stored under anaerobic conditions at 4 °C;
8. vacuum-packaged carrots were treated with AE*Lm*3+*Salmonella enterica* subsp. *enterica* serovar Enteritidis, packaged in polyethylene bags, and stored under anaerobic conditions at 4 °C;
9. vacuum-packaged carrots were treated with AE*Lm*4, packaged in polyethylene bags, and stored under anaerobic conditions at 4 °C;
10. vacuum-packaged carrots were treated with AE*Lm*4+*Salmonella enterica* subsp. *enterica* serovar Enteritidis, packaged in polyethylene bags, and stored under anaerobic conditions at 4 °C.

*Salmonella enterica* subsp. *enterica* serovar Enteritidis (CCM 3807) was used for the antimicrobial effect of AE*Lm* and bacteria were obtained from the Czech Collection of Microorganisms in Brno, Czech Republic. *S*. *enterica* was aerobically cultivated on Tryptone Soya Agar (TSA, Oxoid, Basingstoke, UK) at 37 °C for 24 h. *S*. *enterica* was prepared at 1.5 × 10^8^ CFU (optical density 0.5 McFarland) and added to the sample at a volume of 100 μL. The samples were performed in triplicate.

### 2.3 Samples cultivation

Microbiological tests were carried out on the first and seventh days of storage at 4 °C. 45 mL of 0.1% sterile saline solution was used to dilute 5 g of sample material. The samples were homogenised for 30 min in a shaker (GFL 3031, Burgwedel, Germany). These microbial communities were assessed: Coliform bacteria were identified using Violet Red Bile Lactose Agar (VRBL; Oxoid, Basingstoke, UK) that was cultured at 37 °C for 24–48 h. Plate Count Agar (PCA, Oxoid, Basingstoke, UK) was incubated at 30 °C for 48–72 h to determine the total viable counts. Xylose Lysine Deoxycholate (XLD, Oxoid, Basingstoke, UK) was inoculated with a 0.1 mL sample and incubated for 24 h at 37 °C in order to count the number of *S. enterica*. The media was then evaluated to ascertain the total number of live bacteria, coliforms, and *Salmonella* [21].

### 2.4 Identification of microorganisms by MALDI-TOF MS

Microorganisms isolated from carrot samples were identified using the MALDI-TOF (matrix-assisted laser desorption/ionization time of flight) MS Biotyper (Bruker, Daltonics, Bremen, Germany) based on the comparison of the acquired patterns with reference libraries previously described in the study of Kačániová et al. [21].

### 2.5 Preparation of MALDI matrix solution

A stock solution containing 50% acetonitrile, 47.5% distilled water, and 2.5% trifluoroacetic acid was used as the organic reagent (one mL of the stock solution included 500 μL of pure acetonitrile, 475 μL of pure distilled water, and 25 μL of pure trifluoroacetic acid). A prepared 250 μL of the organic solvent was combined with “HCCA matrix portioned” in an Eppendorf flask. In order to construct the matrix, Brucker (Bremen, Germany) provided all of the ingredients. The method was previously described in the study of Kačániová et al. [21].

### 2.6 Sample preparation and identification

The samples were prepared as previously mentioned by Kunová et al. [22]. Eight colonies on each plate were briefly examined. A Petri dish full of biological material was transferred to an Eppendorf flask along with 300 μL of distilled water, mixed, and 900 μL of ethanol. The mixture was then centrifuged (ROTOFIX 32A, Ites, Vranov, Slovakia) at 10,000 rpm for 2 min. The supernatant was discarded after the precipitate was allowed to dry at 20 °C. The pellet was then treated with 30 μL of 70% formic acid and 30 μL of acetonitrile. The mixture was then centrifuged at 10.000 rpm for 2 min. A MALDI plate was coated with 1 μL of supernatant, which was then followed by the addition of 1 μL of MALDI matrix solution. The samples were dried before being processed for microbe identification in a MALDI-TOF mass spectrometer (Bruker, Daltonics, Bremen, Germany). The Microflex LT MALDI-TOF mass spectrometer (Bruker Daltonics, Bremen, Germany) was used to automatically produce mass spectra with a mass range of 2000-20.000 Da. The Bruker bacterial test standard was used to calibrate the equipment. The MALDI Biotyper 3.0 program (Bruker Daltonics, Bremen, Germany) was used to process the results of the mass spectra. According to the identification criteria, a score of 2.300–3.000 indicated highly probable identification on the species level; a score of 2.000–2.299 secured genus identification with probable species identification; a score of 1.700–1.999 indicated probable identification to the genus level; and a score of less than 1.700 was regarded as unreliable identification.

### 2.7 Statistical analysis

One-way ANOVA, followed by the Tukey's HSD test at *p* < 0.05 significance, was performed using online Astatsa Anova One Way.

## 3 Results and discussion

The aqueous extract of *L. maritima* displays significantly high values of both total phenolic content (TPC) and total flavonoid content (TFC). With a TPC of 325.15 ± 7.77 mg GAE/g and a TFC of 87.44 ± 6.9 mg QE/g, it exhibits significant concentrations of phenolic compounds and flavonoids. These results suggest that the aqueous extraction method effectively solubilizes and concentrates bioactive compounds from *L. maritima*. The high TPC and TFC values indicate a potential for strong antioxidant activity in the AE, making it a promising candidate for further investigation in pharmaceutical and nutraceutical applications.

LC-MS analysis identified several phenolic acids and flavonoids in the AE. Phenolic acids such as protocatechuic acid (C_7_H_6_O_4_), chlorogenic acid (C_16_H_18_O_9_), quinic acid (C_7_H_12_O_6_), *p*-coumaroylmalic (C_13_H_12_O_7_), and cinnamic acid (C_9_H_8_O_2_) as well as flavonoids such as catechin (C_15_H_14_O_6_), myricetin (C_15_H_10_O_8_), naringenin (C_15_H_12_O_5_) and apigenin 7-*O*-glucoside (C_12_H_20_O_10_) were detected according the result obtained in our previous published work [21] (data not shown).

Minimal non-thermal processing techniques, which do not alter the characteristics of the original food product but only marginally restrict the growth of undesirable microbiota, are used to prolong the shelf-life of foodstuffs and ensure their good microbiological quality [23,24]. Unfortunately, the danger of negative changes in the microbiota composition of food items cannot be eliminated by using these approaches, which accelerates the processes of food degradation [25].

A previous study evaluated the chemical composition of identical *L. maritima* extracts with the concept of evaluating antimicrobial activity on beef samples [17]. In our study, day 1. was used to analyse the number of bacteria in the control, in the group treated with *L. maritima* extracts (AE*Lm*), and in the group inoculated with *S. enterica*. The coliform bacteria ranged between 1.23 in the group of AE*Lm*4 (1.25 mg/mL) to 1.52 log CFU/g in control group. The number of *Salmonella* ranged from zero in the untreated group with *S. enterica* to 1.56 log CFU/g in treated group with *S. enterica*. The total count of bacteria varied between 1.35 in the treatment group of AE*Lm*3+SE (2.5 mg/mL) to 1.67 log CFU/g in control group (Table 1).

**Table 1:** Number of different group of organisms on carrot samples after *Salmonella enterica* treatment 1 day (log CFU/g).

Treated group	Coliforms	*Salmonella*	Total count of bacteria	Isolated species of bacteria
Control without vacuum	1.52 ± 0.07^a^	ND	1.67 ± 1.15^a^	*Pseudomonas marginalis, Pseudomonas taetrolens, Pseudomonas veronii, Pseudomonas extremorientalis, Pseudomonas proteolytica, Serratia proteamaculans, Rahnella aquatilis, Rhizobium radiobacter*
Control + vacuum	1.45 ± 1.15^a^	ND	1.59 ± 0.09^a^	*Pseudomonas chlororaphis, Serratia liquefaciens, Serratia fonticola, Raoultella terrigena, Lelliottia amnigena, Pseudomonas rhodesiae, Pseudomonas libanensis, Pseudomonas thivervalensis, Pseudomonas koreensis, Pantoea agglomerans, Rhizobium radiobacter, Rahnella aquatilis, Pseudomonas protegens, Erwinia persicina*
AE*Lm*1	1.34 ± 0.09^a^	ND	1.45 ± 1.15^a^	*Pseudomonas chlororaphis, Raoultella terrigena, Serratia plymuthica, Pseudomonas extremorientalis, Rahnella aquatilis, Serratia liquefaciens, Pseudomonas koreensis, Erwinia persicina, Pantoea agglomerans, Pseudomonas kilonensis, Pseudomonas thivervalensis*
AE*Lm*1+SE	1.37 ± 1.21^a^	1.56 ± 1.12^a^	1.43 ± 1.13^a^	*Pseudomonas koreensis, Raoultella terrigena, Pseudomonas marginalis, Serratia plymuthica, Pseudomonas proteolytica, Klebsiella oxytoca, Salmonella enterica, Kluyvera intermedia, Pantoea agglomerans, Rahnella aquatilis*
AE*Lm*2	1.32 ± 0.10^a^	ND	1.42 ± 1.17^a^	*Raoultella terrigena, Kluyvera intermedia, Pseudomonas corrugata, Klebsiella oxytoca*
AE*Lm*2+SE	1.36 ± 1.23^a^	1.45 ± 1.12^a^	1.51 ± 1.34	*Salmonella enterica, Pseudomonas cedrina, Bacillus cereus, Rahnella aquatilis, Raoultella terrigena, Klebsiella oxytoca*
AE*Lm*3	1.24 ± 0.09^a^	ND	1.44 ± 1.45^a^	*Pseudomonas veronii, Raoultella terrigena, Pseudomonas proteolytica, Pantoea agglomerans, Pseudomonas extremorientalis, Acinetobacter johnsonii, Pseudomonas thivervalensis, Pseudomonas brassicacearum, Rahnella aquatilis, Kluyvera intermedia*
AE*Lm*3+SE	1.29 ± 1.12^a^	1.47 ± 0.09^a^	1.35 ± 1.34^a^	*Serratia liquefaciens, Rahnella aquatilis, Bacillus cereus, Pseudomonas chlororaphis, Pseudomonas extremorientalis, Acinetobacter calcoaceticus, Kluyvera intermedia, Pseudomonas koreensis, Pseudomonas proteolytica, Pseudomonas libanensis, Pantoea agglomerans, Salmonella eneterica, Erwinia persicina*
AE*Lm*4	1.23 ± 0.08^a^	ND	1.42 ± 1.12^a^	*Pseudomonas libanensis, Pseudomonas tolaasii, Pseudomonas fluorescens, Kluyvera intermedia, Pseudomonas chlororaphis, Pseudomonas kilonensis, Pseudomonas koreensis, Raoultella ornithinolytica, Pseudomonas poae, Raoultella terrigena, Rahnella aquatilis*
AE*Lm*4+SE	1.27 ± 1.18^a^	1.38 ± 1.12^a^	1.53 ± 1.41^a^	*Pseudomonas extremorientalis, Serratia proteamaculans, Pseudomonas kilonensis, Pseudomonas brassicacearum, Pseudomonas chlororaphis, Pseudomonas tolaasii, Erwinia rhapontici, Raoultella terrigena, Salmonella enterica, Rahnella aquatilis*

Data are the mean (±SD) of 3 samples. Different letters in each column refer to significant differences (Tukey, *p* ≤ 0.05).

In the study of Määttä et al. [26], washed whole unpeeled carrots had the highest average levels of aerobic plate counts (5.5 log CFU/g). None of the samples showed the presence of *Escherichia coli*. *Pantoea agglomerans* and *Pseudomonas extremorientalis* were the most isolated species in our samples. Enterobacteriaceae and coliforms increased during processing. According to a previous investigation, the initial total bacterial count in minimally processed carrots on sticks increased during processing from 4.7 to 7.0 log CFU/g [27]. Freshly sliced carrot slices with milder slicing technique showed less physical damage and lower microbiological load [28]. Total bacterial counts in sliced carrots were high, averaging approximately 7 log CFU/g [29] or 6 log CFU/g [30]. These results are different from those obtained in our study where the number of microorganisms was lower.

AE*Lm* has antimicrobial effect against bacterial community and especially against *S. enterica*. Phenolic compounds present mainly among plant secondary metabolites have recently aroused great interest as physiologically active chemicals with various health effects. In addition, they can be used instead of synthetic preservatives [31]. Plants rich in polyphenols are known to have additional antibacterial properties [[32], [33], [34]]. In the work of Hsouna et al. [35], Gram-positive and Gram-negative bacteria were tested against *L. maritima* extract. In the study, among the microorganisms investigated, human pathogens were the most prominent, which are known to be opportunists to humans and animals and cause food contamination and spoilage. Many foodborne disorders, in addition to those caused by direct infection, are caused by the presence of toxins released by bacteria.

**Fig. 1:**
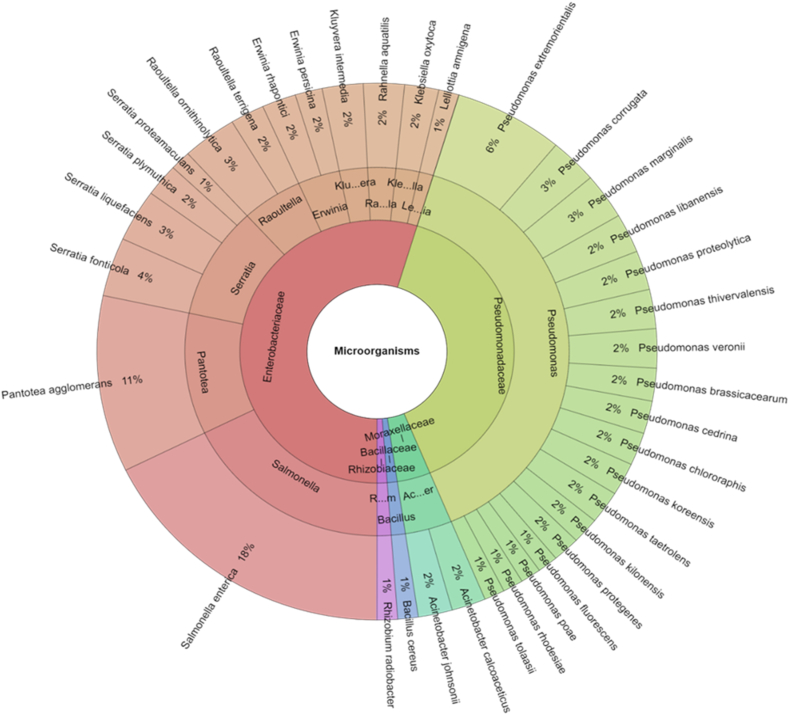
Krona chart: Isolated species of microorganisms from carrot samples after 1st day.

For the better understanding the isolated species of microorganisms with Krona chart in first day are showed Fig. 1. Total 254 isolates were identified and 5 family, 13 genera and 36 species were isolated from carrot samples. The most isolated species was *Salmonella enterica* (17.72%), which was added to samples. The other most isolated bacterial species were *Pantoea agglomerans* (10.63%) and *Pseudomonas extremorientalis* (6.30%).

In the study of Morka et al. [36], *Escherichia coli, Pseudomonas aeruginosa, Staphylococcus aureus, Erwinia* spp., *Enterobacter* spp., *Salmonella* spp., and *Shigella* spp. were among the seven bacterial species detected. *Shigella* spp. (8%) had the lowest rate of incidence, with *E. coli* (20%) and *S. aureus* (15%) showed higher prevalence. In our study, for the first time, an inoculated species from carrots was identified by mass spectrometry.

The most isolated species of bacterial species in our study were *Pseudomonas* spp. Possible human pathogens as well as species associated with decay were found on carrots that were infected with compost. However, numerous *Pseudomonas* species were found on the surface of carrot roots, which are considered to be important in the rotting of peeled packaged carrots [37]. The compost used for the investigation may have provided the carrots with microorganisms. Due to their pectinolytic abilities, species of the genus *Pseudomonas* play an important role in plant diseases and postharvest [38].

Low-temperature refrigeration has been used to store minimally processed fruits and vegetables, extending their shelf life and limiting microbial growth, although it is only effective against psychrotrophic bacteria. Some authors have claimed that lettuce, cabbage, and chicory contain mesophile and psychrotrophic bacteria [39,40].

The number of bacteria in the control, in the group treated with *L. maritima* extract, and in the group inoculated with *S. enterica* were analysed on day 7. of our investigation. The treated group's coliform bacteria levels varied from 1.38 log CFU/g in the group treated with AE*Lm*3 (2.5 mg/mL) to 1.68 log CFU/g in the control group. In the untreated group where not *Salmonella* sp. was not identified to 1.67 log CFU/g in the treated group with *Salmonella* and AE*Lm*1+*SE* (10 mg/mL), the number of *Salmonella* varied. In the treatment group, the total bacterial count ranged from 2.23 log CFU/g in the group with treatment of AELm3 (2.5 mg/mL) o 2.69 log CFU/g in control group without vacuum packaging (Table 2).

**Table 2:** Number of different groups of organisms on carrot samples after *Salmonella enterica* treatment 7 day (log CFU/g).

Treated group	Coliforms	*Salmonella*	Total count of bacteria	Isolated species of bacteria
**Control without vacuum**	1.68 ± 0.12^a^	ND	2.69 ± 1.12^a^	*Serratia liquefaciens, Pantoea agglomerans, Lelliottia amnigena, Pseudomonas orientalis, Pseudomonas libanensis, Pseudomonas koreensis*
**Control** + **vacuum**	1.63 ± 1.16^a^	ND	2.57 ± 0.32^a^	*Serratia plymuthica, Rahnella aquatilis, Serratia liquefaciens, Ewingella americana, Pantoea agglomerans, Erwinia persicina, Raoultella terrigena*
**AE*Lm*1**	1.45 ± 1.34^a^	BD	2.37 ± 0.39^a^	*Serratia liquefaciens, Raoultella terrigena, Serratia plymuthica, Pseudomonas extremorientalis, Lelliottia amnigena, Enterobacter ludwigii, Pantoea agglomerans, Enterobacter cloacae*
**AE*Lm*1** + **SE**	1.57 ± 1.43^a^	1.67 ± 1.26^a^	2.45 ± 0.39^a^	*Salmonella enterica, Serratia quinivorans, Serratia plymuthica, Erwinia rhapontici, Erwinia persicina, Pseudomonas marginalis,*
				*Enterobacter ludwigii, Raoultella terrigena, Serratia liquefaciens, Erwinia persicina, Lelliottia amnigena, Pseudomonas trivialis, Rahnella aquatilis, Pseudomonas chlororaphis, Pantoea agglomerans, Raoultella terrigena, Enterobacter cloacae, Enterobacter ludwigii*
**AE*Lm*2**	1.46 ± 1.45^a^	ND	2.36 ± 0.39^a^	
**AE*Lm*2** + **SE**	1.51 ± 0.34^a^	1.57 ± 1.23^a^	2.43 ± 0.49^a^	*Salmonella enterica, Klebsiella oxytoca, Raoultella terrigena, Erwinia persicina, Kluyvera intermedia, Erwinia rhapontici, Serratia liquefaciens, Lelliottia amnigena, Serratia plymuthica, Pantoea agglomerans*
**AE*Lm*3**	1.38 ± 1.78^a^	ND	2.23 ± 0.40^a^	*Raoultella planticola, Serratia liquefaciens, Raoultella terrigena, Enterobacter cloacae, Kluyvera intermedia, Serratia plymuthica, Pantoea agglomerans*
**AE*Lm*3** + **SE**	1.43 ± 1.34^a^	1.55 ± 0.15^a^	2.28 ± 0.30^a^	*Serratia liquefaciens, Serratia plymuthica, Enterobacter asburiae, Raoultella ornithinolytica, Rahnella aquatilis, Erwinia rhapontici, Pseudomonas cedrina, Pseudomonas veronii, Raoultella terrigena, Pantoea agglomerans, Enterobacter ludwigii, Salmonella enterica*
**AE*Lm*4**	1.51 ± 0.09^a^	ND	2.32 ± 0.25^a^	*Raoultella terrigena, Enterobacter cloacae, Serratia liquefaciens, Serratia plymuthica, Rahnella aquatilis, Erwinia rhapontici, Aeromonas media, Pantoea agglomerans*
**AE*Lm*4** + **SE**	1.55 ± 1.24^a^	1.45 ± 1.25^a^	2.43 ± 0.39^a^	*Enterobacter ludwigii, Salmonella enterica, Serratia liquefaciens, Raoultella terrigena, Serratia plymuthica, Erwinia rhapontici, Erwinia persicina, Pseudomonas tolaasii, Pseudomonas grimontii, Pseudomonas antarctica, Pantoea agglomerans, Raoultella terrigena, Enterobacter cloacae*

Data are the mean (±SD) of 3 samples. Different letters in each column refer to significant differences (Tukey, *p* ≤ 0.05).

In this study by Pilon et al. [41], the count of psychrotrophic in green pepper and carrot after storage that had undergone little processing ranged from 10^3^ to 10^6^ CFU/g and 10^2^–10^5^ CFU/g, respectively. The number of microorganisms in our study was significantly lower 10^3^ CFU/g than those discovered by García-Gimeno & Zurera-Cosano [42], who noted an increase of 10^5^–10^7^ CFU/g in the number of psychrotrophic microorganisms in mixed lettuce, carrot, and purple cabbage salad packed in polypropylene film without gas injection for 9 days of storage at a temperature of 4 °C. With initial indices of 10^6^–10^9^ CFU/g, minimally processed carrots, beetroot, and mixed salad from several commercial brands had substantial concentrations of psychrotrophic bacteria [43]. The sanitary quality of minimally processed vegetables was studied by several authors, such as on mixed cabbage, carrot, onion and green pepper salad [44], on cauliflower and spinach [45], on cabbage [46], on wild chicory [47], on chicory [39] on celery [48], on lettuce [49], on mixed lettuce, green pepper and cucumber salad [50] and on garlic [51] and in general, the products are safe when processed in hygiene conditions and stored under refrigeration temperatures. Considering that our study agrees with previous results, we can assume that vacuum packing and sharpening of the aqueous extract of *L. maritima* affected the number of microorganisms.

**Fig. 2:**
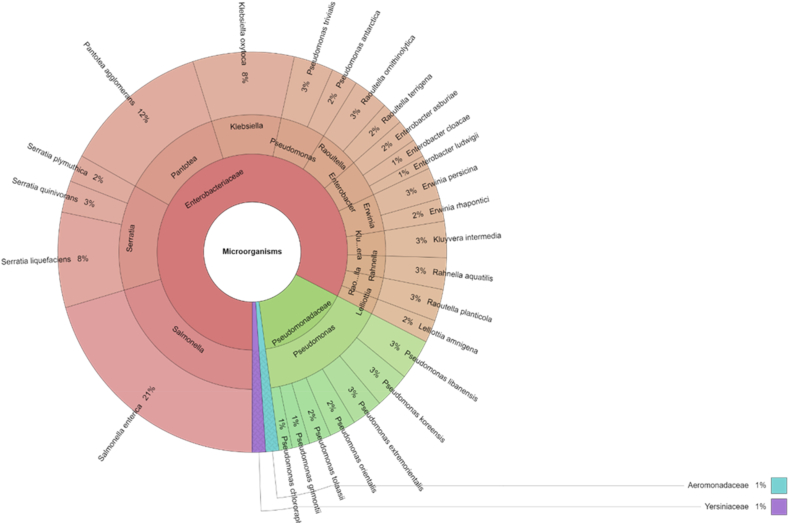
Krona chart: Isolated species of microorganisms from carrot samples after 7th day.

The species of microorganisms isolated over 7 days are shown in Fig. 2. Total of 273 isolates were identified and 4 family, 13 genera and 28 species were isolated from carrot samples. The most isolated species was *Salmonella enterica* (20.51%), which was added to samples. Other more isolated bacterial species were *Pantoea agglomerans* (12.09%) and *Klebsiella oxytoca* (6.30%). Most of the species isolated from carrot after seven days were similar to the species isolated after the first day *Pseudomonas* spp.

While *Yersinia* spp. are associated with disease outbreaks, psychotropic strains of the genera *Pseudomonas, Erwinia*, and *Enterobacter* are frequently linked to the post-harvest spoiling of chilled vegetables [52]. The higher amount of Proteobacteria, which was up to 34% higher on washed carrots stored at 10 °C compared to 2 °C, was responsible for the reduced term and uniformity on carrots stored at 10 °C. In particular, the relative abundance of *Pseudomonas* and *Janthinobacterium* was 17% higher in cleaned carrots stored at 10 °C than in carrots stored at 2 °C. Since some *Pseudomonas* and *Janthinobacterium* species are psychrotrophic and grow faster at 10 °C [53], this is not surprising.

When carrots were maintained at 10 °C for 7 days by all washing methods compared with 2 °C, the number of members of the Firmicutes, especially the Bacillales, was dramatically reduced by 18–21%. Analysis provided additional that washing with disinfectants and storage at low temperature reduced the relative abundance of Enterobacteriaceae. A small (2–5 %) but not statistically significant increase in the relative abundance of Enterobacteriaceae was observed when cleaned carrots were stored at 10 °C compared with 2 °C [54]. In other studies, a slight increase in the relative abundance of Enterobacteriaceae was observed after 7 days of storage of baby spinach, from 5% to approximately 10–20% at 8 °C and 15 °C [38]. In cleaned baby spinach stored at 4 °C for 15 days compared to 10 °C, members of Enterobacteriaceae were predominant [55]. Coriander essential oil (CEO) has shown promising potential as a novel sustainable washing agent against *S. enterica* on carrot samples, in addition to positive outcomes from *in vitro* investigations. Indeed, this study showed that *Salmonella* populations in artificially infected stick carrots can be effectively reduced for up to one day of storage by using CEO (5 μL/mL) for a brief period of time (2 min). However, the populations on the treated sample increased slightly towards the end of the storage period, and their numbers were the same as in the control sample. These findings not only demonstrate the ability of *Salmonella* spp. to survive and multiply at low temperatures but also the ability to adapt to the stress conditions induced by the CEO [56]. In this context, Kalily et al. [57] showed how *S. senftenberg* adapted to linalool, which also gave other antimicrobial treatments improved protection. In contrast to our findings, Ndoti-Nembe et al. [58], found no significant reduction of the *S. typhimurium* load at day 1 of treatment with savory EO (0.35% v/v), however a reduction of roughly 2 log CFU/g was demonstrated by treatment with savory EO after 9 days on chilled mini carrots.

## 4 Conclusions

The aim of our study was to determine the antimicrobial effect of an aqueous extract of *L. maritima* on the microbiological quality of vacuum-packed carrot slice samples treated with four different concentrations and inoculated with *S. enterica*, a bacteria that poses a safety risk in the consumption of foods such as carrots. Our results showed that a concentration of 2.5 mg/mL had a positive effect on the abundance of microorganisms on carrot slice samples. We can also conclude that the optimum concentration used in our study can eliminate the number of *S. enterica* bacteria at the lowest concentrations when combined with vacuum packing. We can also conclude that the optimum concentration used in our study can eliminate the number of *S. enterica* bacteria at the lowest concentrations in combination with vacuum packing. Our results also showed that plant aqueous extracts at optimal concentration can be used for the shelf life of foods such as carrots from a microbiological point of view.

## Funding

This research was funded by the grant APVV-20-0058 “The potential of the essential oils from aromatic plants for medical use and food preservation”.

## Data availability statement

The datasets generated for this study are available on request to the corresponding author.

## CRediT authorship contribution statement

**Anis Ben Hsouna:** Writing – original draft, Validation, Software, Resources, Investigation, Formal analysis, Conceptualization. **Natália Čmiková:** Writing – review & editing, Writing – original draft, Visualization, Validation, Software, Methodology, Investigation, Formal analysis, Data curation, Conceptualization. **Boutheina Ben Akacha:** Writing – original draft, Validation, Software, Resources, Investigation, Formal analysis, Conceptualization. **Rania Ben Saad:** Writing – original draft, Validation, Software, Resources, Investigation, Formal analysis, Conceptualization. **Wissem Mnif:** Writing – original draft, Validation, Software, Resources, Investigation, Formal analysis, Conceptualization. **Stefania Garzoli:** Writing – review & editing, Writing – original draft, Visualization, Validation, Software, Resources, Investigation, Formal analysis, Conceptualization. **Miroslava Kačániová:** Writing – review & editing, Writing – original draft, Visualization, Validation, Supervision, Software, Resources, Project administration, Methodology, Investigation, Funding acquisition, Formal analysis, Data curation, Conceptualization.

## Declaration of competing interest

The authors declare that they have no known competing financial interests or personal relationships that could have appeared to influence the work reported in this paper.
